# Key role for inhibins in effective T cell activation, migration and Th17 differentiation

**DOI:** 10.1002/2211-5463.70106

**Published:** 2025-08-29

**Authors:** Sandra Ortega‐Francisco, Roxana Olguín‐Alor, Lizbeth Bolaños‐Castro, Alexandra Morales‐Cruz, Marisol De La Fuente‐Granada, Gloria Soldevila

**Affiliations:** ^1^ Departamento de Immunología Instituto de Investigaciones Biomédicas, Universidad Nacional Autónoma de México Mexico; ^2^ Laboratorio Nacional de Citometría de Flujo Instituto de Investigaciones Biomédicas, Universidad Nacional Autónoma de México Mexico

**Keywords:** activation, inhibins, migration, T cells, Th1/T17

## Abstract

Our group has previously reported that inhibin and its molecular pair, TGF‐β type III receptor (TβRIII), regulate T cell development within the thymus. In addition, inhibins play a key role in immune tolerance through the modulation of dendritic cell (DC) maturation and peripheral Treg induction. However, the functional role of inhibins in T cell activation and differentiation is currently unknown. Here, we demonstrate that inhibins are produced by activated T cells and play a role during T cell activation, migration, and functional Th differentiation. Specifically, stimulation of Inhα^−/−^ naïve T cells resulted in decreased expression of early activation markers, including CD69, CD25, and TβRIII, compared to Inhα^+/+^ T cells. Additionally, we analyzed the migratory potential of Inhα^−/−^ T cells toward CCR7 ligands and showed an impaired *in vitro* chemotaxis toward CCL21 and CCL19, which correlated with a decreased homing to peripheral lymph nodes using *in vivo* competitive assays. To evaluate the impact of inhibins on Th differentiation, we performed *in vitro* polarization cultures under skewing conditions. Interestingly, Inhα^−/−^ naïve T cells showed a decreased differentiation to Th1 cells, while the induction of Th17 cells was significantly increased when compared with Inhα^+/+^. This preferential polarization of Inhα^−/−^ toward Th17 was reversed by the addition of recombinant Inh A, without altering the differentiation of Inhα^−/−^ Th1. Our data demonstrate that inhibins regulate T cell effector differentiation and homing and thus, may be considered new players in T cell immune responses.

## Graphical abstract

Activins/inhibins modulate immune responses. Here, we show that inhibins can be produced very early after T cell activation and can act in an autocrine way, favoring Th1 versus Th17 differentiation and CCL19/CCL21 mediated migration. Recombinant inhibin A can restore the Th1/Th17 balance, highlighting the role of inhibins as key regulators of T cell function and immune homeostasis.

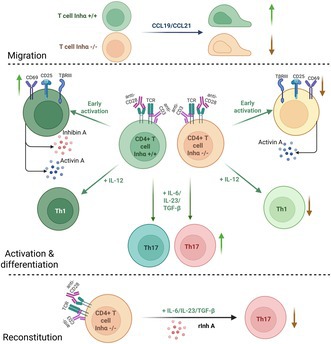

**Abbreviations**
BMPs: bone morphogenetic proteins
DC: dendritic cell
DTH: delayed‐type hypersensitivity
EAE: experimental autoimmune encephalomyelitis
MLN: mesenteric lymph nodes
PLN: peripheral lymph nodes
SP: spleen
TCR: T‐cell receptor
TβRIII: TGF‐β type III

Effective T cell‐mediated responses require optimal activation, migration to lymphoid organs, and effector T cell differentiation (reviewed in [1, 2]). Through different effector or regulatory subtypes, T cells orchestrate the polarization of the immune response by the secretion of Th‐derived cytokines [3].

There is growing evidence that several TGF‐β superfamily members are involved in the modulation of the immune response [4]. Specifically, TGF‐β plays a key role in T cell tolerance, promoting regulatory T cell induction and downregulating antigen‐presenting cell function [5, 6]. In the context of effector T cell differentiation, TGF‐β inhibits Th1 and Th2 polarizations but promotes Th9, Th17, and Tfh [7]. In addition, bone morphogenetic proteins (BMPs) have recently been described to inhibit the differentiation of naïve CD4^+^ T cells into Th17 cells while promoting iTreg cell differentiation [8]. Finally, activins were initially described as Th2 cytokines [9, 10], although later studies demonstrated that, similar to TGF‐β, they can also promote Treg [11, 12, 13], Th17 [14, 15], Th9 [16], and Thf [17] differentiation. However, to date, no reports have yet investigated the role of inhibins on T cell‐mediated immune responses.

Activins and inhibins were initially described as hormones with antagonizing functions in several endocrine processes [18, 19]. The inhibin–receptor complex antagonizes the Activin signaling pathway through the association of inhibin β subunit to the activin type II receptor (ActRII) and the binding of inhibin α subunit to the TGFβ receptor III (Betaglycan) [20].

Our group has studied inhibins as key immune system modulators, both during T cell development and T cell activation. We demonstrated that inhibins play a role in specific thymocyte selection checkpoints [21, 22]. In addition, we showed that inhibins regulate thymic stromal development and maturation, influencing thymic Treg generation [23]. Finally, we characterized that inhibins also impact peripheral DC maturation and function, as Inhα^−/−^ DCs exhibit an impaired capacity to stimulate allogeneic T cell responses and delayed‐type hypersensitivity (DTH) responses, which were associated with a reduced migratory capacity both *in vitro* and *in vivo* [24]. In contrast, Inhα^−/−^ mDCs displayed an enhanced capacity to induce CD25^+^ Foxp3^+^ regulatory T cells indicating their potential role in peripheral T cell tolerance [25].

Recently, we reported that TβRIII is expressed in CD4^+^ T cells and becomes upregulated after polyclonal T‐cell receptor (TCR) stimulation. Interestingly, TβRIII appears to play an important role during *de novo* induction of induced Tregs (iTregs) [26]. Moreover, the role of TβRIII in effector T cells was recently explored by our group, using conditional mice lacking TβRIII on peripheral T cells [27], demonstrating that this receptor plays an important role in Th17/Th1 pathogenicity in the experimental autoimmune encephalomyelitis (EAE) model.

Given the growing evidence indicating that inhibins and TβRIII may act as molecular partners for different biological processes, including thymocyte selection [22, 28] and endocrine‐mediated functions [29], we postulated that inhibins could also be involved in T cell activation and differentiation.

Here, we investigated the role of inhibins in CD4^+^ T cell activation, migration, and Th1 versus Th17 differentiation and identified these ligands as potential mediators of T cell immune responses.

## Materials and methods

### Mice

Inhibin α deficient mice (Inhα^−/−^) in C57BL/6 background were generated and kindly donated by Dr. Martin Matzuk (Baylor College of Medicine, Houston, TX, USA). Four‐week‐old female mice were used in all experiments. All animals were maintained in specific pathogen‐free (SPF) conditions at the “Unidad de Modelos Biológicos” of the “Instituto de Investigaciones Biomédicas (IIB, UNAM, Mexico).” All animal handling and experimental procedures were done according to the ethics guidelines. The study was approved by the “Comité para el Cuidado y Uso de Animales de Laboratorio (CICUAL)” of the Institute under protocol #176.

### Evaluation of CD4 ^+^ T subpopulations in homeostasis

Inhα^−/−^ and Inhα^+/+^ CD4^+^ T cells from the spleen (SP), peripheral lymph nodes (PLN—including axillary and popliteal lymph nodes), and mesenteric lymph nodes (MLN) were analyzed by flow cytometry to evaluate the percentage and total number of naïve (CD44^lo^ CD62L^hi^ CCR7^+^), central memory (CD44^hi^ CD62L^+^ CCR7^+^), and effector memory (CD44^hi^ CD62L^−^ CCR7^−^) subpopulations. In addition, we evaluated the levels of TβRIII and CCR7 expression among all these subpopulations. 1 × 10^6^ cells from each organ were stained, and FMO controls were used as controls for gating.

### *In vitro* T cell activation and differentiation

**Fig. 1:**
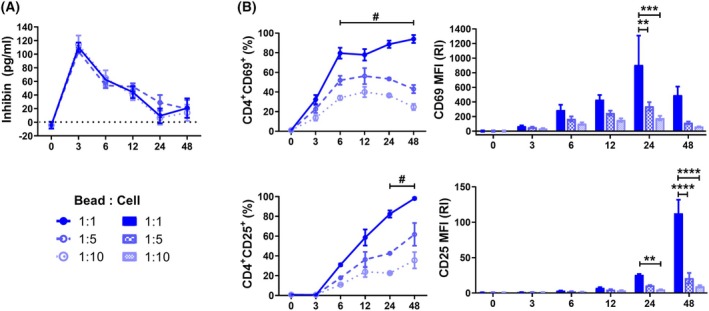
Inhibin A production after polyclonal activation is independent of TCR strength. (A) Inhibin A concentration was detected in the supernatants of wild‐type naïve T cells after 3, 6, 12, 24, and 48 h of activation with anti‐CD3/anti‐CD28 beads, using different bead‐to‐cell proportions (1 : 1; 1 : 5; 1 : 10). Graphs show the concentration in pg·mL^−1^. The dotted line represents the detection limit. (B) The percentages of positive cells (left) and the relative expression levels (right) of CD69 and CD25 were analyzed in Inhα^+/+^ naïve CD4^+^ T cells upon activation. MFI was determined and reported as relative increment (RI) compared to time zero. Graphs show mean ± SEM. Statistical significance was determined by a two‐way ANOVA test. *P ≤ 0.05, **P ≤ 0.01, ***P ≤ 0.001, ****P ≤ 0.0001. #: data were statistically different at 1 : 1, 1 : 5, and 1 : 10 bead‐to‐cell proportions. *n* = 3 for all conditions.

**Fig. 2:**
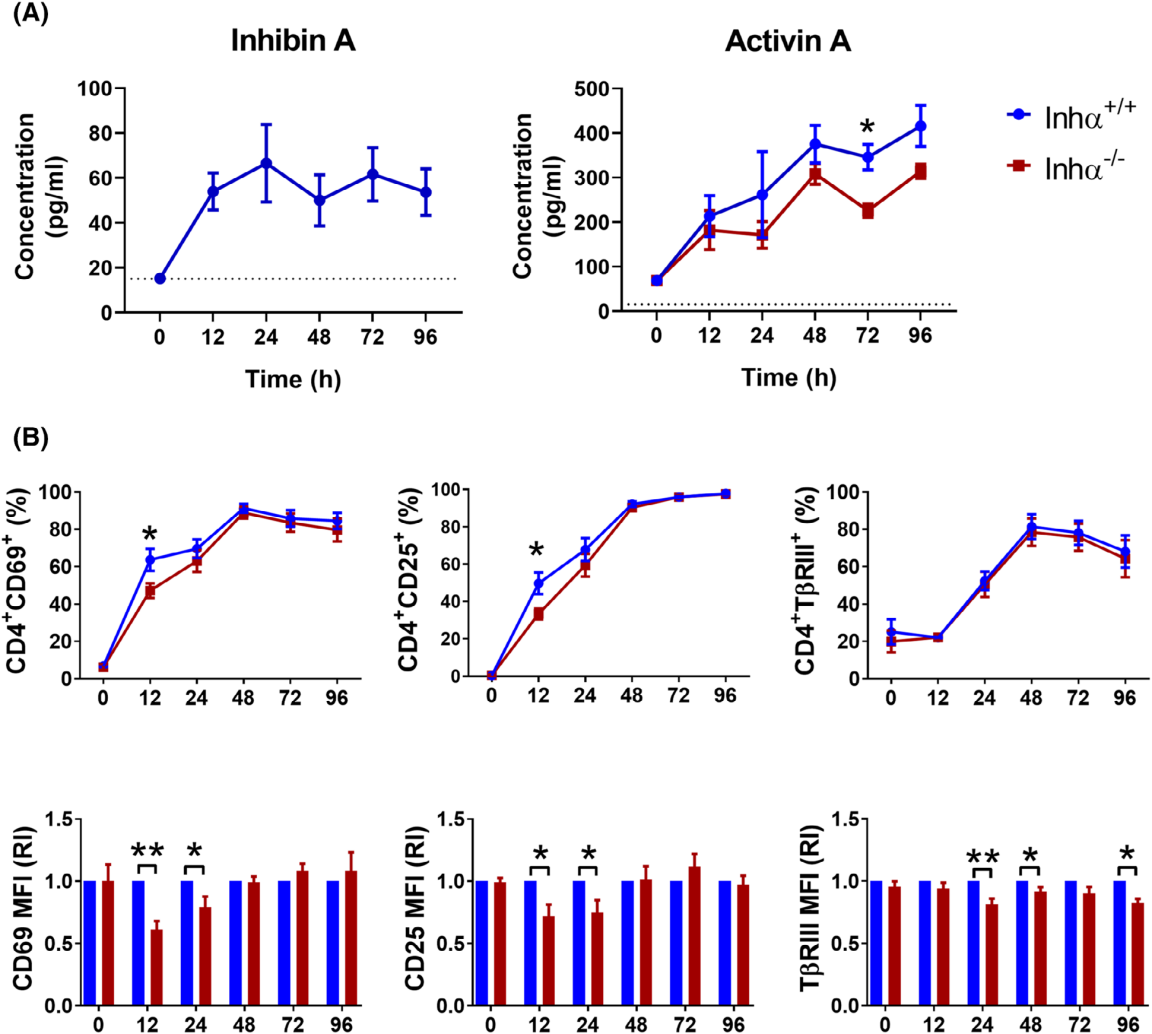
Inhibins are required for optimal CD4^+^ T cell activation. (A) Inhibin A (left) and Activin A (right) production were measured in the supernatants of Inhα^+/+^ or Inhα^−/−^ naïve CD4^+^ T cell cultures after 12, 24, 48, and 96 h of activation with plate‐bound anti‐CD3 (1 μg·mL^−1^) and anti‐CD28 (1 μg·mL^−1^). Graphs show the concentration in pg·mL^−1^, with the dotted line showing the detection limit. (B) The percentages of positive cells (top) and the relative expression levels (bottom) of CD69, CD25, and TβRIII were analyzed in Inhα^+/+^ and Inhα^−/−^ naïve T cells upon activation. MFI was determined and reported as relative increment (RI) compared to Inhα^+/+^ mice. Graphs show mean ± SEM. Statistical significance was determined by a two‐tailed unpaired *t*‐test. *P ≤ 0.05, **P ≤ 0.01. *n* = 3–4 for all conditions.

CD4^+^ CD25^−^ CD44^lo^ CD62L^hi^ naϊve T cells were sorted from spleen and peripheral lymph nodes obtained from Inhα^−/−^ and Inhα^+/+^ mice by flow cytometry (FACS Aria I cell sorter BD Biosciences, San Jose, CA, USA, Becton & Dickinson, Franklin Lakes, NJ, USA, Moflo Astrios cell sorter, Miami, FL, USA, Beckman Coulter, Brea, CA, USA) and cultured for as long as 4 days with either anti‐CD3/anti‐CD28 beads at different proportions to cells (1 : 1, 1 : 5 or 1 : 10) or plate‐bound anti‐CD3 (1 μg·mL^−1^) and anti‐CD28 (1 μg·mL^−1^) antibodies in complete medium. Activation markers (CD69, CD25, CD44, and TβRIII) from live CD4^+^ cells were evaluated at 3, 6, 12, 24, 48, 72, and 96 h, depending on the experiment. Mean fluorescence intensities (MFI) of the activation markers were expressed as relative increments (RI) and calculated as: MFI stimulation time/MFI time zero (Fig. 1) or MFI of Inhα^−/−^ cells / MFI of Inhα^+/+^ cells (Fig. 2).

For T cell differentiation, sorted naïve T cells were first labeled with CTV and incubated for 3 days with plate‐bound anti‐CD3 (1 μg·mL^−1^) and anti‐CD28 (1 μg·mL^−1^) antibodies in complete medium in the presence of polarizing conditions, as previously described [27]. For inhibin reconstitution experiments, 100 pg·mL^−1^ of recombinant mouse inhibin (R&D Systems, Minneapolis, MN, USA) was added to the corresponding wells. The induction of regulatory T cells (Tregs) was carried out using different TGF‐β concentrations (0.5, 1.0, and 2.5 ng·mL^−1^). Th1, Th17, and Treg cells were analyzed, and the proliferation indexes were calculated, as previously described [27].

### Inhibin and activin production

Inhibin A secretion was evaluated from the supernatants of Inhα^+/+^ naïve T cells at different times after activation (0–96 h), while the production of Activin A was analyzed in the supernatants of both Inhα^−/−^ and Inhα^+/+^ naïve T cells at the same time points. Activin A and Inhibin A were quantified using the Human/Mouse/Rat Activin A Quantikine ELISA Kit (R&D Systems), Mouse InhA ELISA Kit (Elabscience, Houston, TX, USA) (15 pg·mL^−1^ detection limit), and Mouse Inhibin A ELISA Kit (MyBioSource, San Diego, CA, USA) following the instructions of the manufacturer.

### *In vitro* T cell migration

Migration assays were performed as previously described [30]. Briefly, calcein‐labeled lymph node cells (1 × 10^5^ cells per well) were placed in a modified Boyden chamber with increasing concentrations (0–1000 nm) of CCL9 and CCL21. After 2 h, transmigrated cells were quantified using a Typhoon FL 9500 Scanner and analyzed with the imagequant tl software. The chemotaxis index was calculated by dividing the fluorescence of migrated cells by the fluorescence of cells without chemokine stimulation (chemokinesis).

### *In vivo* T cell migration

Competitive homing assays were performed as previously described [30]. Briefly, peripheral lymph node cells from Inhα^−/−^ and Inhα^+/+^ mice were labeled with CTV and CFSE, respectively. 5 × 10^6^ cells were resuspended in 50 μL PBS, mixed at 1 : 1, and injected intravenously into C57BL/6 mice. After 18 h, the lymphocyte homing was assessed by evaluating the total number of cells that reached the lymph nodes related to the infused lymphocytes for each subpopulation (CD4^+^, CD8^+^, or CD19^+^). All data were normalized after calculating the ratio Inhα^−/−^/ Inhα^+/+^ (CTV : CFSE) of cells present in each lymphoid organ compared to that of the original transferred mix.

### Flow cytometry

Flow cytometry staining was performed as previously described [26]. For intracellular staining, cells were fixed and permeabilized by using the Foxp3/Transcription Factor Staining Buffer kit (Tonbo Biosciences, San Diego, CA, USA) and then incubated with the intracellular antibodies. All samples were acquired in an Attune Nxt cytometer (Thermofisher, Waltham, MA, USA) and analyzed with the flowjo 10 software (BD Biosciences, San Jose, CA, USA). Table S1 shows the list of antibodies and dyes used.

### Statistics

All data are represented as mean ± SEM. For the statistical analysis, 2‐way ANOVA or two‐tailed *t*‐test analysis was performed using graphpad prism 8.0.2. When an ANOVA indicated significant differences, a Tukey's test was used for multiple comparisons of individual means. *P ≤ 0.05; **P ≤ 0.01; ***P ≤ 0.001; ****P ≤ 0.0001.

## Results and discussion

### T cells produce inhibin a after polyclonal stimulation

It has been described that T cells produce several ligands of the TGF‐β superfamily upon activation. For instance, TGF‐βs, BMPs, and activins are secreted by T cells following TCR crosslinking [9, 31, 32]. Here, we report the production of Inhibin A after anti‐CD3/anti‐CD28 activation of naïve T cells. Inhibin production showed an early peak at 3 h post activation (110.14 ± 6.84 pg·mL^−1^, 1 : 1), which was moderately maintained after 48 h of stimulation (20.733 ± 14.3 pg·mL^−1^, 1 : 1) (Fig. 1A). Furthermore, we demonstrated that the production of inhibin A is not dependent on the strength of the activation stimulus, since using different proportions of anti‐CD3/anti‐CD28 beads : cells showed the same levels of Inhibin A production, despite T cells upregulating their activation markers in a dose–response manner (Fig. 1B and Fig. S1A).

We next performed plate‐bound anti‐CD3 and anti‐CD28 stimulation and evaluated both inhibin A and activin A production in Inhα^+/+^ and Inhα^−/−^ mice, as it has been reported that the absence of inhibins can result in overproduction of activins [33].

As shown in Fig. 2, activin A was readily produced after T cell stimulation, even at higher levels than inhibins, detecting 213.6 ± 65.3 pg·mL^−1^ at 12 h after stimulation and reaching the highest amount at 96 h (416.2 ± 65 pg·mL^−1^). Interestingly, Inhα^−/−^ T cells secreted lower levels of activin A at 72 h after activation compared to Inhα^+/+^ T cells (Fig. 2A). This was an unexpected result, as one of the mechanisms proposed for inhibin‐mediated antagonism of activin‐mediated functions is the competition for the β subunits during dimer formation, which nevertheless correlates with the reported increase of Activins in the sera of Inhα^−/−^ mice [24, 33].

In summary, as T cell stimulation was performed in the absence of antigen‐presenting cells, we propose that the intrinsic production of Inhibin A in activated T cells could play a role in T cell‐mediated immune responses.

### Naïve Inhα^−/−^ T cells are increased in peripheral lymph nodes under homeostatic conditions

As we have previously shown that inhibins regulate T cell differentiation in the thymus [21, 23], we decided to explore whether they also might be involved in peripheral T cell biology. To evaluate the effect of inhibins in CD4^+^ T cell homeostasis, we analyzed both the percentage and total numbers of naïve, central memory, or effector memory subpopulations from PLN, MLN, and spleen of Inhα^−/−^ compared to Inhα^+/+^ mice. We used 4‐week‐old mice to avoid the potential effects of increased systemic activin levels, as well as the presence of gonadal tumors, which develop in older Inhα^−/−^ mice [24, 33]. As shown in Fig. S1, the absence of inhibins leads to an increase in total CD4^+^ T cells and CD4^+^ naïve T cells in PLN but not in other lymphoid organs (spleen and MLN), while, as expected, both central and effector memory cells were not affected as mice have not previously been challenged. These data suggest a potential effect of inhibins in CCR7‐mediated homing or viability of migrated cells. The fact that under homeostatic conditions, we observed increased numbers of total cells in Inhα^−/−^ PLN compared to Inhα^+/+^ mice could potentially be due to increased levels of CCR7 and CD62L on Inhα^−/−^ cells in this tissue (Fig. S1B), which could partially compensate for the decreased chemotaxis toward CCR7 ligands of these cells.

Interestingly, in a recent report, we have demonstrated that the conditional deletion of TβRIII in CD4^+^ T cells (T*gfbr3fl/fl.dLcKCre* mice) does not affect the homeostatic homing of CD4^+^ T cells to any of the lymphoid organs analyzed [27], suggesting that not all inhibin‐mediated functions are associated with TβRIII expression or function. In this context, we did not observe any differences in the levels of the TβRIII coreceptor on Inhα^−/−^ T cells compared to Inhα^+/+^ T cells (data not shown), confirming that it is preferentially expressed on central memory and naïve CD4^+^ T cells (not shown) as previously reported [26].

### Inhα^− /−^ T cells show impaired activation after TCR crosslinking

Although the *ex vivo* central memory and effector memory cell subpopulations proportions were not affected by the absence of inhibins, based on recent data by our group demonstrating that TβRIII (a molecular pair for inhibins) modulates T cell activation and Th effector function [27] and given our data showing that T cells can intrinsically produce inhibins upon T cell activation, we hypothesized that inhibins might also be involved in TCR‐mediated signaling. As before, sorted naïve T cells were stimulated, and the expression of activation markers was evaluated. Interestingly, we observed that the expression of the early activation markers CD25 and CD69 at 12 and 24 h following stimulation (Fig. 2B and Fig. S2B) was significantly lower on Inhα^−/−^ T cells than ono Inhα^+/+^ T cells. On the other hand, TβRIII expression was significantly lower in Inhα ^−/−^ T cells at 24, 48, and 96 h of stimulation compared to Inhα^+/+^ T cells (Fig. 2B). Altogether, these data suggest that inhibins may be involved in achieving optimal T cell activation or favoring a more sustained activated phenotype.

The molecular signals by which inhibins may promote T cell activation have not yet been elucidated; although unpublished data by our group showed the activation of Erk1/2 in mouse thymocytes after stimulation with both InhA and Inh B. In agreement with these findings, it was reported that inhibins/activins signaling can lead to Erk phosphorylation in human gonadal and adrenal cancers [34].

### Inhα^−/−^ T cells show impaired *in vitro* and *in vivo* migration

**Fig. 3:**
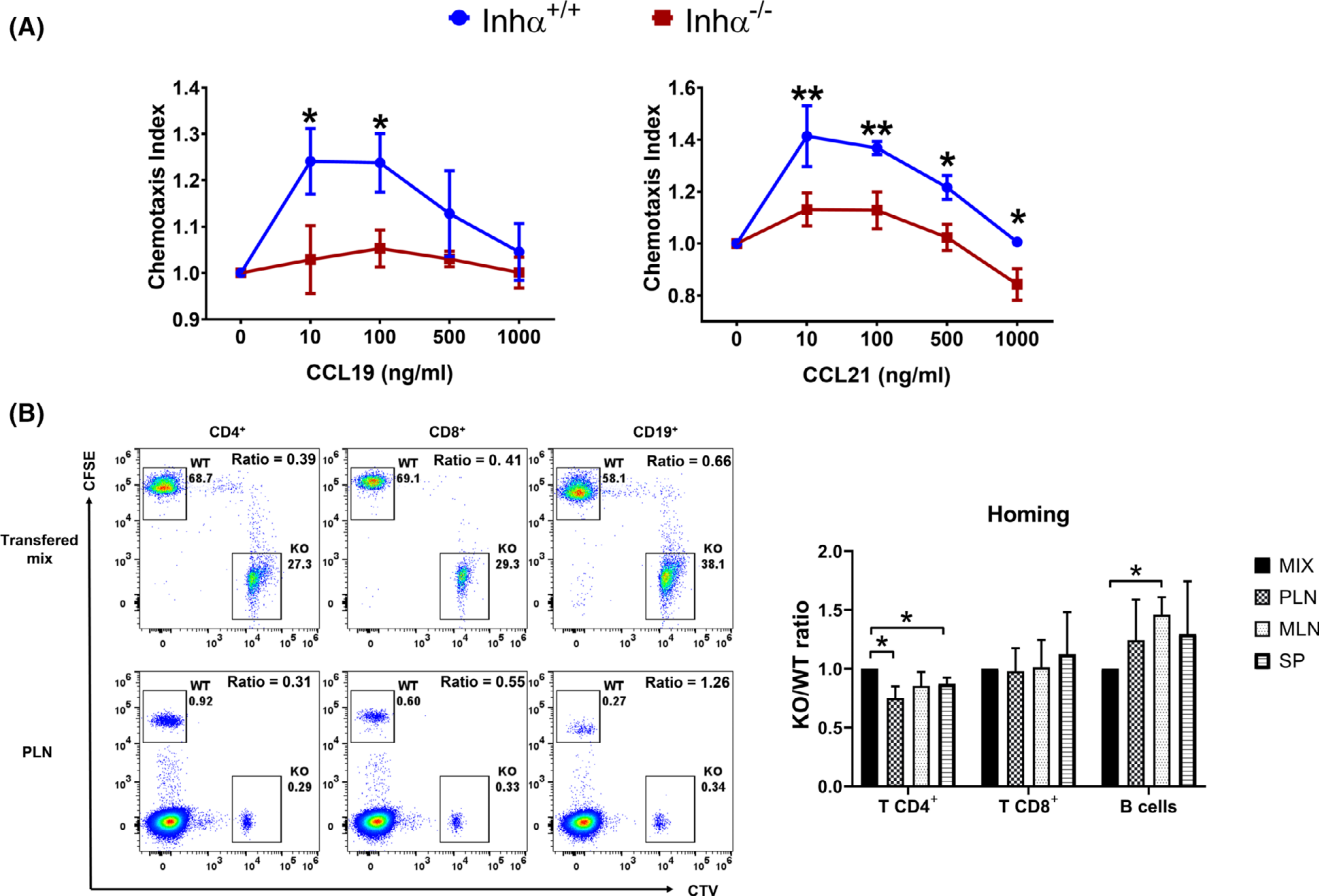
Inhα^−/−^ T cells show impaired *in vitro* and *in vivo* migration toward CCR7 ligands. (A) The chemotaxis index of Inhα^+/+^ and Inhα^−/−^ lymphocyte cells were evaluated at different concentrations of CCL19 (left) and CCL21 (right). (B) A mix (1 : 1) of Inhα^+/+^ (CFSE^+^) and Inhα^−/−^ (CTV^+^) lymphocytes were inoculated (i.v.) into C57BL/6 mice, and the *in vivo* migration to lymph organs was analyzed by measuring the percentage of transferred cells present in SP, LN, or MLN. Representative dot plots from transferred mix and transferred cells at PLN are shown (left). Bar graphs (right) show the Inhα^−/−^ / Inhα^+/+^ ratio of CD4^+^ T, CD8^+^ T or B CD19^+^ cells. All data were normalized after calculating the ratio Inhα^−/−^/ Inhα^+/+^ (CTV : CFSE) of cells present in each lymphoid organ compared to that of the original transferred mix. Graphs show mean ± SEM. Statistical significance was determined by a two‐tailed unpaired *t*‐test. *P ≤ 0.05, **P ≤ 0.01. *n* = 3–4 for all conditions.

T cell homing is a key process both for the maintenance of homeostasis as well as for the development of effector immune responses. Here, we performed *in vitro* chemotaxis assays to analyze the migratory capacity of Inhα^−/−^ or Inhα^+/+^ cells toward the CCR7 ligands, CCL19 and CCL21. We observed that Inhα^−/−^ cells have a significantly decreased chemotaxis index toward both ligands at different chemokine concentrations compared with Inhα^+/+^ T cells (Fig. 3A). In addition, we performed *in vivo* homing assays and analyzed percentages and total numbers of cells that migrated to PLN, MLN, and SP 18 h after adoptive transfer into nonirradiated recipient mice. Our data showed that Inhα^−/−^ CD4^+^, but not CD8^+^ or CD19^+^ cells, present an impaired migration to lymph nodes compared to Inhα^+/+^ T cells (Fig. 3B and Fig. S3), demonstrating a key role of inhibins in T lymphocyte migration *in vivo*.

This decreased homing does not correlate with the enhanced CCR7 levels observed in T cells from Inhα^−/−^ PLN under homeostatic conditions (Fig. S1); instead, our data suggest that the increased total numbers of CD4^+^ T cells could be the result of alterations in other homing receptors, including integrins and selectins, or LN retention signals such as S1P1R (reviewed in [35, 36]).

### Inhα^−/−^ T cells show decreased *in vitro* Th1 differentiation and an increase in Th17 compared to Inhα^+/+^ T cells

As the development of an effective immune response depends both on optimal T activation and migration, we hypothesized that the differences observed in Inhα^−/−^ T lymphocytes could influence Th differentiation.

**Fig. 4:**
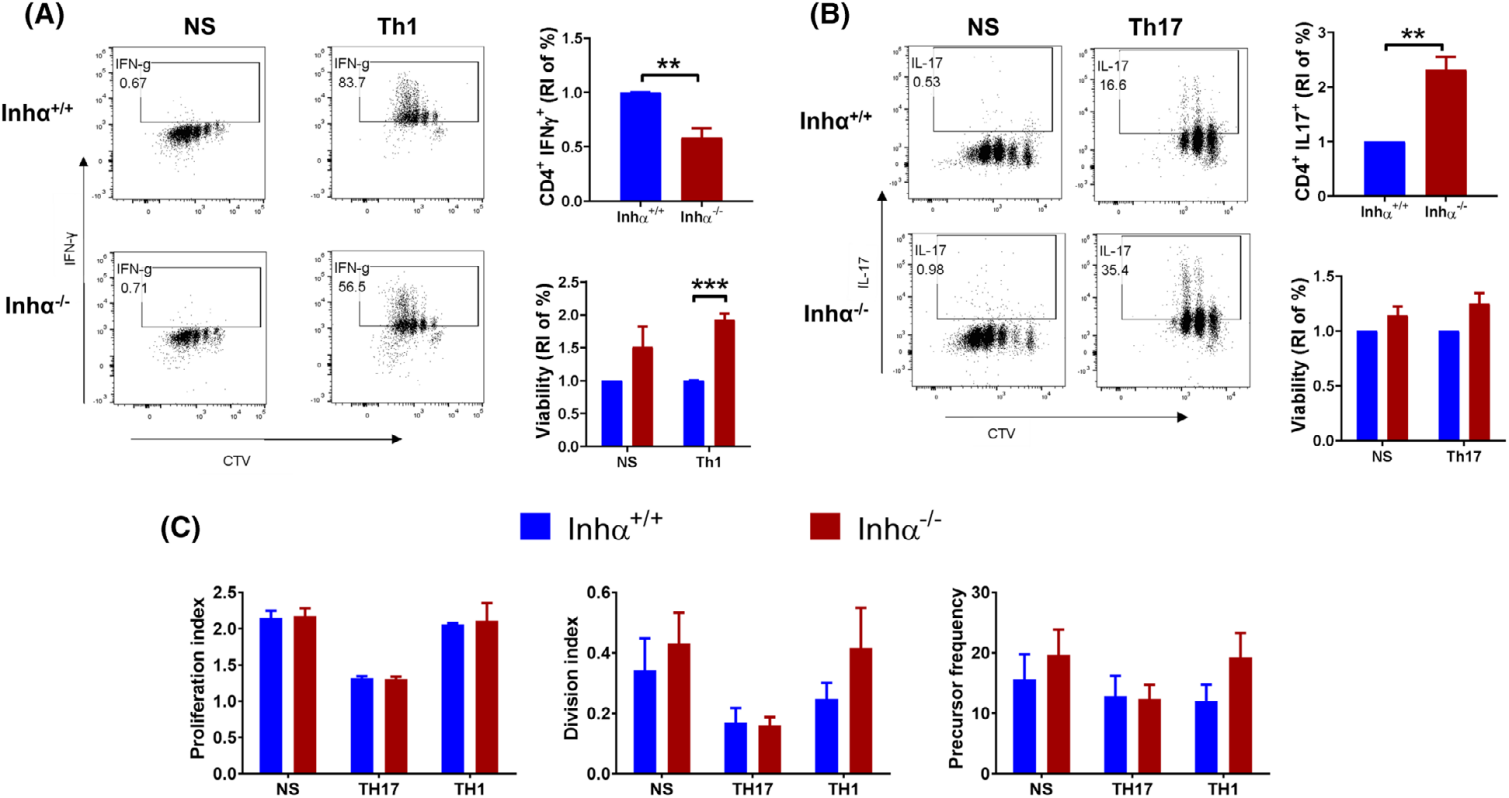
Inhα^−/−^ CD4^+^ T show a decreased Th1 but an increased Th17 differentiation. Inhα^−/−^ and Inhα^+/+^ naïve CD4^+^ T cells were cultured under polarizing Th1, Th17 or non‐skewing conditions (NS). (A) Th1 differentiation, representative dot plots (CTV vs. IFNγ) showing the proliferation and differentiation of Inhα ^+/+^ (top) and Inhα ^−/−^ (bottom) Th1 cells. Bar graphs show the relative percentage of Inhα^−/−^ CD4^+^ IFNγ^+^ (top) and live cells (bottom), compared to Inhα^+/+^. (B) Th17 differentiation, representative dot plots (CTV Vs IL‐17) showing the proliferation and differentiation of Inhα^+/+^ (top) and Inhα ^−/−^ (bottom) Th17 cells. Bar graphs show the relative percentage of Inhα^−/−^ CD4^+^ IL‐17^+^ cells (top) and live cells (bottom), compared to Inhα^+/+^. (C) Proliferation index, division index, and precursor frequency were obtained from the same cultures. Graphs show mean ± SEM. Statistical significance was determined by a two‐tailed unpaired *t*‐test. *P ≤ 0.05, **P ≤ 0.01, ***P ≤ 0.001. *n* = 3.

To evaluate the intrinsic effect of inhibins on Th differentiation, we cultured naïve T cells under Th1 and Th17 polarizing conditions. As shown in Fig. 4, we observed a significant decrease in the differentiation (RI = 0.58 ± 0.089) of Inhα^−/−^ cells toward the Th1 lineage when compared to its wild‐type counterpart. In contrast, Th17 differentiation was significantly increased (RI = 2.3 ± 0.23) in Inhα^−/−^ T cells in comparison with Inhα^+/+^. One possibility that could account for these differences is that the TCR signal threshold required for Th1 is higher than for Th17 differentiation [37].

In this context, our Inhα^−/−^ T cells displayed a decreased expression in early T activation markers at 12 h of stimulation (Fig. 1), which could influence Th cell fate. In agreement with this, IFNγ production appears to be downregulated in Inhα^−/−^ Th1 cells as early as 24 h after stimulation (during the first division cycle), compared to Inhα^+/+^. Alternatively, the enhanced differentiation of Th17 may be the result of different responsiveness to TGF‐β; although when we evaluated the *in vitro* induction of Inhα^−/−^ Tregs from naïve T cells, we observed no significant differences in the percentage of iTregs generated using different TGF‐β concentrations compared to Inhα^+/^, although increased total numbers were observed possibly due to enhanced viability (Fig. S4), arguing against this possibility.

Unexpectedly, the decreased Th1 polarization of Inhα^−/−^ T cells was accompanied by a significant increase in Th cell viability under these skewing conditions. This increase in viability was also the case for Inhα^−/−^ iTregs, where a significant increase in total iTreg number was detected (Fig. S4). In contrast, we only observed a trend toward an increase in the viability of Th17 differentiated Inhα^−/−^ T cells, and likewise in Inhα^−/−^ T cells activated under non‐skewing conditions, suggesting that the increased viability can be cytokine related.

### Recombinant inhibin counteracts the enhanced Th17 differentiation of Inhα^−/−^ T cells

**Fig. 5:**
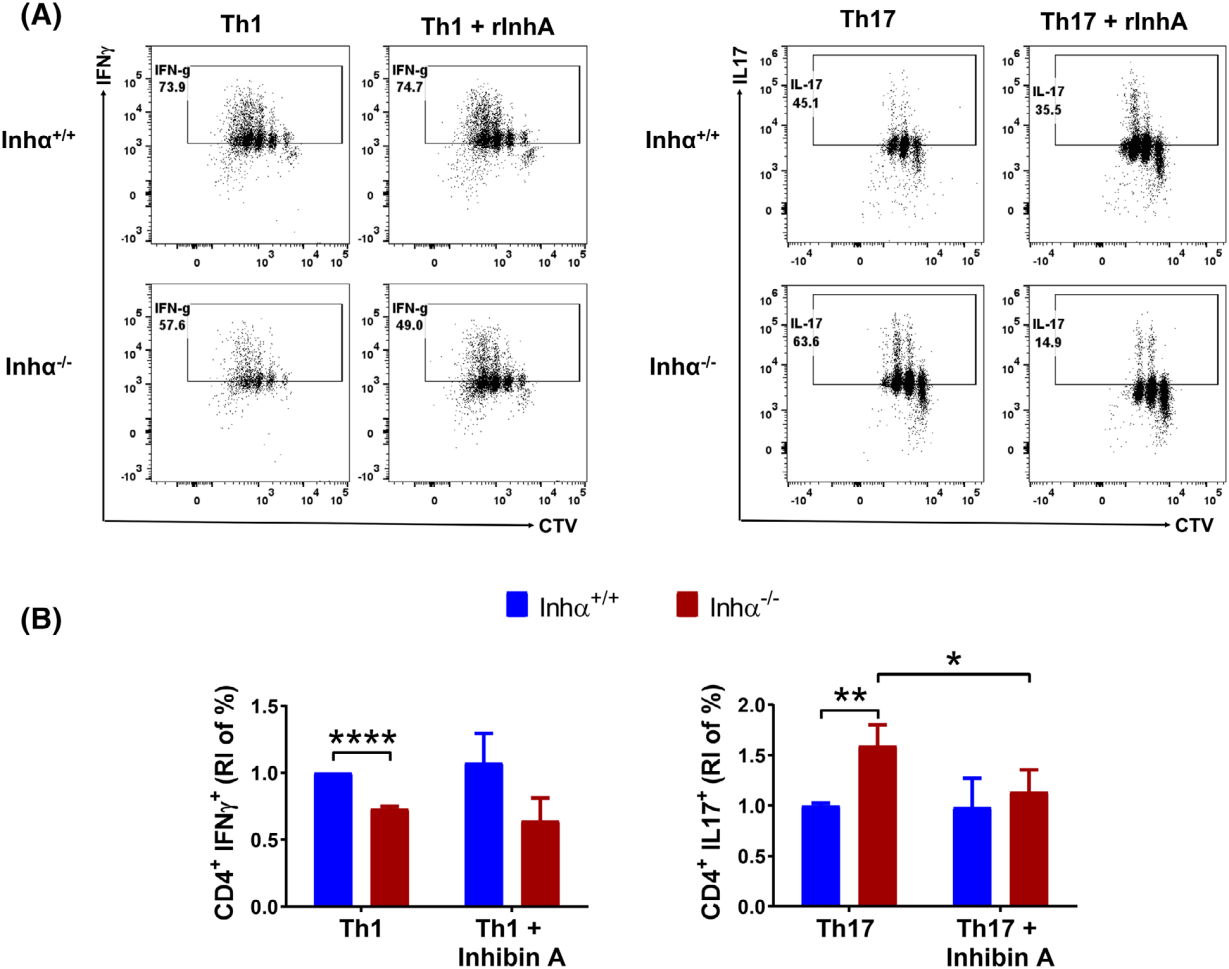
Exogenous inhibin A decreases Inhα^−/−^ CD4^+^ Th17 cell differentiation without affecting Th1 differentiation. Inhα^−/−^ and Inhα^+/+^ naïve CD4^+^ T cells were cultured under Th1, Th17, and non‐skewing conditions (NS) in the presence or absence of recombinant mouse Inhibin A (100 pg·mL^−1^). (A) Representative dot plots from Th1 (left) and Th17 (right) differentiation of Inhα^+/+^ (top) and Inhα^−/−^ (bottom) are shown. (B) Bar graphs show the relative increment (RI) of the percentage of Inhα^−/−^ CD4^+^ IFNγ^+^ T cells (right) and CD4^+^ IL‐17^+^ T cells (left) compared to Inhα^+/+^ cells. Graphs show mean ± SEM. Statistical significance was determined by a two‐tailed unpaired *t*‐test. *P ≤ 0.05, **P ≤ 0.01, ****P ≤ 0.0001. *n* = 3.

As our differentiation cultures were performed in the absence of antigen‐presenting cells, we investigated the potential autocrine role of inhibins in Th effector differentiation. For this, we cultured naïve T cells under Th1 or Th17 skewing conditions in the presence of recombinant murine inhibin A (rInh A) (100 pg·mL^−1^). Interestingly, rInh A was able to partially reverse the increased Th17 polarization of Inhα^−/−^ T cells compared to Inhα^+/+^ T cells, reducing the levels of Th17 differentiation to those observed in Inhα^+/+^ T cells (RI = 1.6 to RI = 1.13 ± 0.5) (Fig. 5). In contrast, the addition of rInh A did not result in significant changes in Th1 differentiation of neither deficient nor sufficient Inhα T cells.

Activins have been involved in the modulation of several inflammatory processes (reviewed in [38]). Although we observed lower Activin production after TCR stimulation of Inhα^−/−^ T cells, compared to Inhα^+/+^ T cells, we cannot exclude the possibility that Activin expression may be upregulated under polarizing conditions, as Activins have been reported to synergize with TGF‐β in Tregs conversion [11]. However, we did not observe an increased iTreg generation, which argues against this possibility. In addition, it has been reported that under Th17 polarizing conditions, transcription of the activin Type 2 receptor gene (Acvr2) is upregulated, suggesting that activins may favor Th17 polarization [39]; furthermore, activin produced during an autoimmune or inflammatory process promotes Th17 differentiation [40].

## Conclusions

Inhibins influence T cell development, activation, and effector differentiation through mechanisms involving modulation of TCR‐dependent signals, cytokine responsiveness, and chemokine‐dependent migration. Specifically, we propose that inhibins promote effective T cell activation and migration to lymphoid organs and favor the differentiation of Th1 cells while restraining Th17 differentiation. This evidence further supports the role of inhibins as key immune system regulators.

## Conflict of interest

The authors declare no conflict of interest.

## Author contributions

GS, SO‐F, and RO‐A contributed to the conception and design of the study. SO‐F, LB‐C, AM‐C, and MDLF‐G performed the experiments; SO‐F and RO‐A analyzed the data; GS, SO‐F, RO‐A, and MDLF‐G contributed to the interpretation of the data. SO‐F wrote the original draft of the article; RO‐A, MDLF‐G, and GS contributed writing, reviewing, and editing the manuscript; all authors approved the final version.

## Supporting information


**Table S1.** List of flow cytometry reagents.

**Fig. S1.** Evaluation of Inh⍺^−/−^ CD4^+^ T subpopulations under steady‐state conditions.

**Fig. S2.** Expression of activation markers (CD69, CD25, and TβRIII) after CD4+ T cell activation.

**Fig. S3.** *In vivo* migration assay.

**Fig. S4.** *In vitro* Treg differentiation.

## Data Availability

The data that support the findings of this study are available from the corresponding author soldevi@iibiomedicas.unam.mx upon reasonable request.
