# Supplementary material for: Key role for inhibins in effective T cell activation, migration and Th17 differentiation
Source: FEBS Open Bio. 2025 Aug 29;16(1):79–89. doi: 10.1002/2211-5463.70106 (PMC12767762; doi:10.1002/2211-5463.70106)
Supplement: Supplementary file 1 — Table S1. List of flow cytometry reagents. [file FEB4-16-79-s004.docx]

**Supplementary Table 1. List of Flow Cytometry reagents**

| **Reagent** | **Source (Brand)** | **Identifier (#Catalog)** | **Clone** |
| --- | --- | --- | --- |
| anti-mouse TβRIII | R&D Systems | AF5034 | Polyclonal Goat IgG |
| donkey anti goat IgG (H+L) Cross-Adsorbed Secondary Antibody, AF488 | Invitrogen | A11055 | Polyclonal Donkey IgG |
| anti-mouse CD4 APC | Biolegend | 100412 | GK1.5 |
| anti-mouse CD25 PECy5 | Biolegend | 102010 | PC61 |
| anti-mouse/human CD44 PE | Biolegend | 103008 | IM7 |
| anti-mouse CD62L APC Cy7 | Biolegend | 104428 | MEL-14 |
| anti-mouse CCR7-PE CF594 | BD | 563596 | 4B12 |
| anti mouse CD4 FITC | Biolegend | 100406 | GK1.5 |
| Zombie Aqua | Biolegend | 423101 | – |
| anti-mouse IFNγ BV510 | Biolegend | 505841 | XMG1.2 |
| anti-mouse IL-17A PE | Biolegend | 506904 | TC11-18H10.1 |
| anti-mouse Foxp3 APC | Tonbo Bioscience | 205773 | MF23 |
| anti mouse CD8 PE-Cy7 | Biolegend | 100722 | 53-6.7 |
| anti-mouse CD19 PE | Biolegend | 152407 | 1D3 |
| Zombie NIR | Biolegend | 423106 | – |
