# Supplementary figures and images for: Key role for inhibins in effective T cell activation, migration and Th17 differentiation
Source: FEBS Open Bio. 2025 Aug 29;16(1):79–89. doi: 10.1002/2211-5463.70106 (PMC12767762; doi:10.1002/2211-5463.70106)

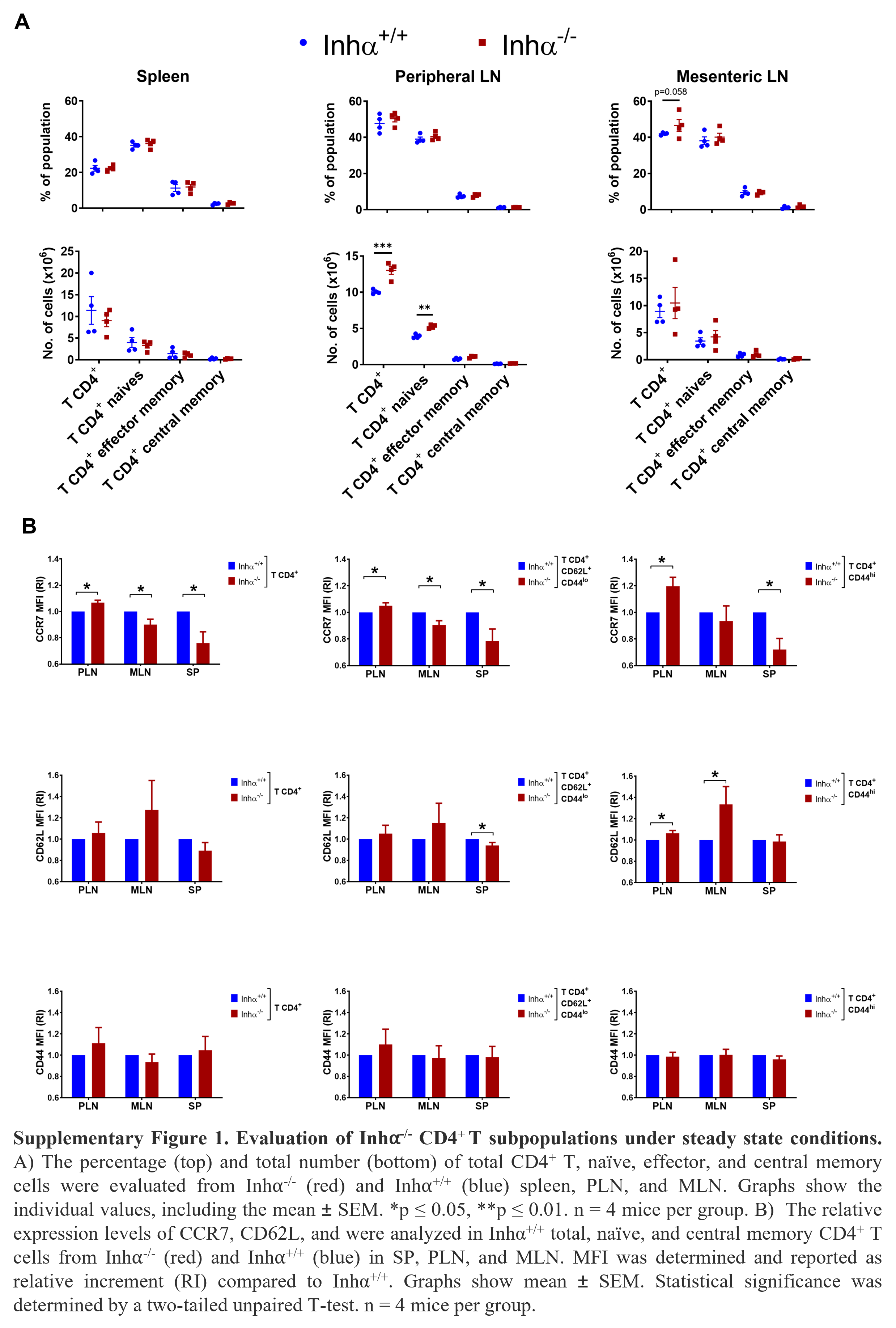

Supplement: Supplementary file 2 — Fig. S1. Evaluation of Inh⍺−/− CD4+ T subpopulations under steady‐state conditions. [file FEB4-16-79-s001.tif]

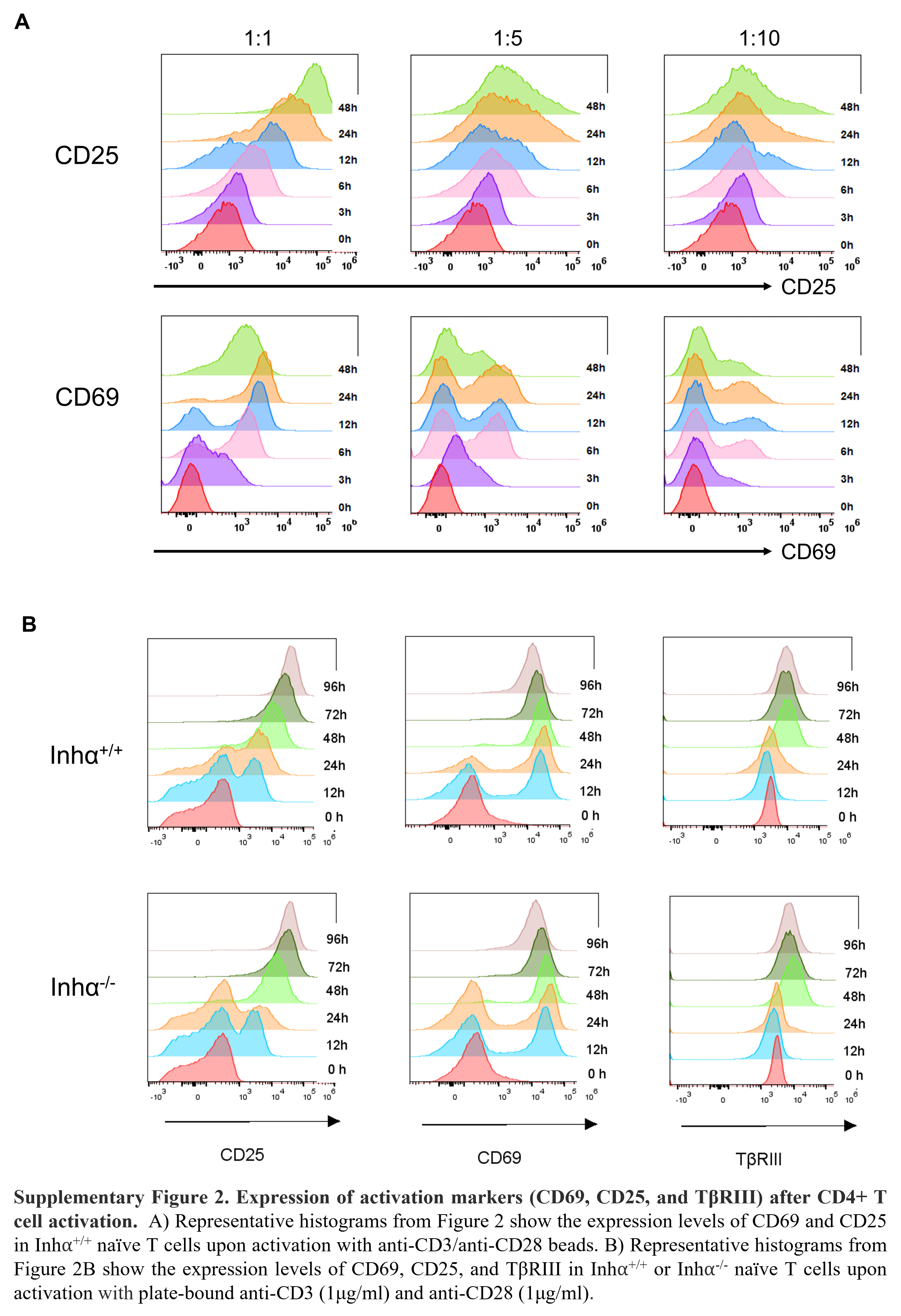

Supplement: Supplementary file 3 — Fig. S2. Expression of activation markers (CD69, CD25, and TβRIII) after CD4+ T cell activation. [file FEB4-16-79-s002.tif]

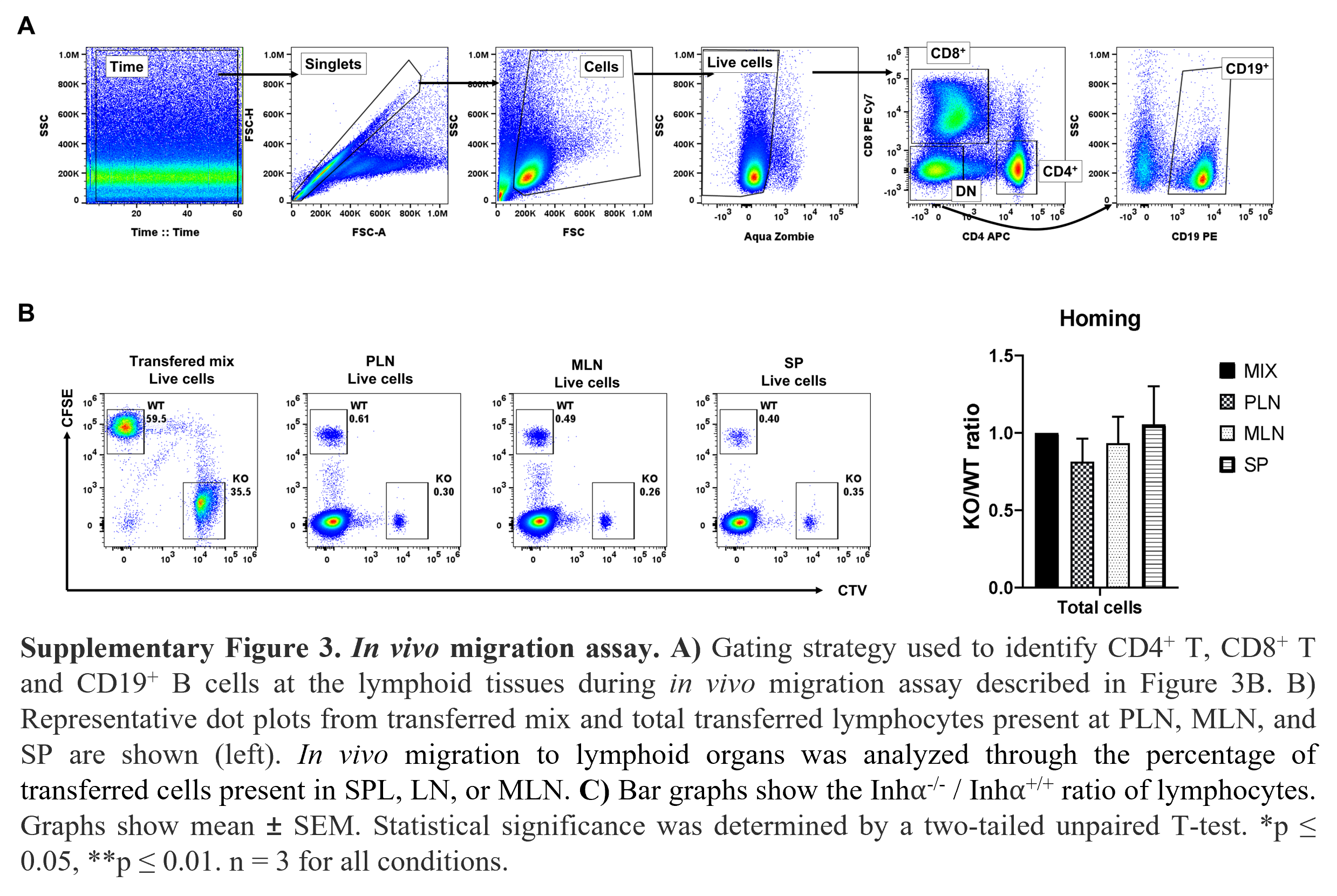

Supplement: Supplementary file 4 — Fig. S3. In vivo migration assay. [file FEB4-16-79-s005.tif]

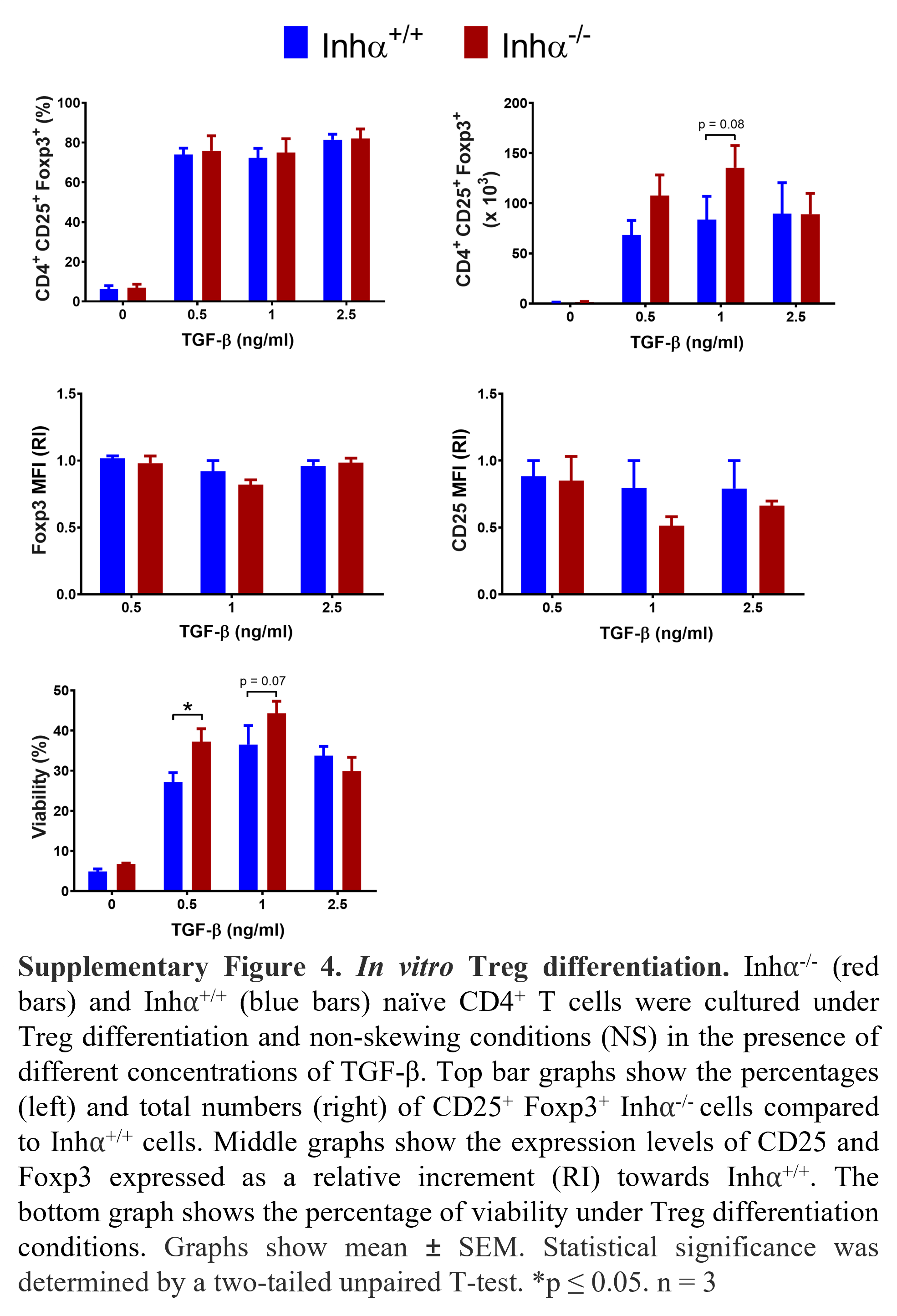

Supplement: Supplementary file 5 — Fig. S4. In vitro Treg differentiation. [file FEB4-16-79-s003.tif]
